# Visual Recovery and Prognosis in the Treatment of Submacular Hemorrhage due to Polypoidal Choroidal Vasculopathy and Retinal Arterial Macroaneurysm: A Retrospective Study

**DOI:** 10.1155/2023/3880297

**Published:** 2023-06-12

**Authors:** Jianhua Wu, Tao Yan, Rui Zhang, Chao Feng, Changzhong Xu, Tao Xu, Yanzi Li, Shan Wang, Junwen He

**Affiliations:** ^1^Department of Retinal & Vitreous Diseases, Aier Eye Hospital of Wuhan University, Wuhan 430000, China; ^2^Department of Ophthalmic Imaging, Aier Eye Hospital of Wuhan University, Wuhan 430000, China

## Abstract

**Purpose:**

This study was carried out to evaluate the visual acuity (VA), complications, and prognosis of patients diagnosed with submacular hemorrhage (SMH) from polypoidal choroidal vasculopathy (PCV) and retinal arterial macroaneurysm (RAM) treated by pars plana vitrectomy (PPV), subretinal tissue plasminogen activator (tPA), and air tamponade in vitreous cavity. It facilitates the development of generic treatment methods that can be widely used to improve vision and treat potential complications in patients with SMH, regardless of the underlying pathophysiological condition, such as PCV or RAM.

**Methods:**

In this retrospective study, SMH patients were divided into two groups based on their diagnosis: (1) polypoidal choroidal vasculopathy (PCV) and (2) retinal arterial macroaneurysm (RAM). The visual recovery and complications of patients with PCV and RAM after PPV + tPA (subretinal) surgery were analyzed.

**Results:**

A total of 36 eyes of 36 patients were included: PCV (47.22%, 17/36) and RAM (52.78%, 19/36). The mean age of the patients was 64 years, and 63.89% of the patients (23/36) were female. The median VA was 1.85 logMAR before surgery, 0.93 and 0.98 logMAR at 1 and 3 months after surgery, respectively, indicating that most patients' vision improved after surgery. At the 1 and 3 months postoperative follow-up, each patient was diagnosed with rhegmatogenous retinal detachment at 1 month and 3 months postoperatively, and four patients had vitreous hemorrhage at 3 months postoperatively. Preoperatively, patients exhibited macular subretinal hemorrhage, retinal bulge, and exudation around the blood clot. Postoperatively, most patients showed dispersal of subretinal hemorrhage. Optical coherence tomography results revealed retinal hemorrhage involving the macula and hemorrhagic bulges under both the neuroepithelium and the pigment epithelium under the fovea preoperatively. After surgery, the air injected into the vitreous cavity was completely absorbed and the subretinal hemorrhage was dispersed.

**Conclusion:**

PPV combined with subretinal tPA injection and air tamponade in the vitreous cavity can facilitate modest visual recovery in patients with SMH due to PCV and RAM. However, some complications may occur, and their management remains challenging.

## 1. Introduction

Submacular hemorrhage (SMH) is a hematoma typically located between the neurosensory retina and the retinal pigment epithelium, but it can also occur beneath the retinal pigment epithelium, and it has several causes [1, 2]. Blood coagulation causes degeneration of red blood cells and the release of iron and hemosiderin, resulting in oxidative stress. Unstable iron is one of the key factors in oxidative damage to cellular proteins, lipids, nucleic acids, and other cellular components [3]. Chemotaxis of macrophages and fibroblasts leads to the release of inflammatory mediators and fibrin, resulting in scar formation. Furthermore, blood clots in the subretinal space adhere to the photoreceptors. Avulsion of the photoreceptors may occur due to contraction of the clot [4, 5].

There is no consensus on the best treatment for SMH secondary to polypoidal choroidal vasculopathy (PCV) [6]. Patients with PCV who develop massive SMH experience severe vision loss. The incidence rate of massive SMH in PCV increases over time [7, 8]. There are many therapeutic options for PCV, including vascular endothelial growth factor (VEGF) injection [9], intravitreal bevacizumab administration combined with pneumatic displacement [10], and pars plana vitrectomy (PPV) + tissue plasminogen activator (tPA) injection [11].

Retinal arterial macroaneurysm (RAM) is an acquired focal dilation of the retinal artery that usually occurs in the first three steps of the retinal artery tree and is commonly seen in older adults with systemic hypertension [12, 13]. Although the prognosis of most patients with RAM is good, multistage retinal hemorrhage due to RAM rupture can lead to severe vision loss, especially when there is macular involvement. Many studies have reported that the visual prognosis of ruptured RAM with SMH is worse than that of RAM with other signs, including premacular or vitreous hemorrhage (VH) and macular edema [14, 15].

The most effective treatments for PCV and RAM include PPV, subretinal tPA, and vitreous air tamponade, which may be due to liquefaction and faster displacement of blood clots [16]. Limitations include greater invasiveness and higher complication rates. Alternatively, intravitreal injection of tPA and air may constitute a less invasive, less complicated choice for patients [17, 18]. This is a minimally invasive approach, and although the risk of complications is low and visual recovery is good, the management of potential complications remains challenging.

In this study, all patients were treated with PPV + subretinal tPA injection and vitreous air tamponade. We chose to evaluate a generic approach that included all patients, regardless of their underlying pathophysiological condition, to derive a broader application of the expected visual outcome and potential complications of the treatment.

## 2. Methods

### 2.1. Patients

A retrospective study was conducted on patients diagnosed with SMH at Aier Eye Hospital of Wuhan University between July 2017 and October 2021. SMH was defined as the accumulation of blood under the area of the macula, which contains the light-sensitive cells in the retina. The diagnosis of PCV was based on the criteria of orange nodular lesions, an abnormal branching choroidal vascular network identified by indocyanine green angiography, and polypoidal choroidal vascular dilatation foci in the subretinal region. The diagnosis of RAM was based on the presence of a grade 2 or 3 vascular bulge of large volume in a large branch of the central retinal artery.

### 2.2. Procedure

In this study, the patients were divided into two groups according to their diagnosis: (1) PCV and (2) RAM. Our hospital recorded the clinical data of patients, including age, sex, disease course, diagnosis, and follow-up period. All patients underwent visual acuity (VA) testing (best-corrected Snellen visual acuity, BCVA) and optical coherence tomography (OCT, RTVue XR, Optovue, Inc., Fremont, CA, USA) scans using the RTVue XR software (version 2018.1.0.43). Fundus photographs were taken with an Optos PLC panoramic ophthalmoscope (200TX, Dunfermline, UK) using the Optos V^2^®Vantage Pro Review software (version 2.11.0.3). The subretinal injections of the tissue plasminogen activator (tPA) were performed using the 38 G needle head (PolyTip® Cannula 23 G/38 G (2 mm); MedOne Surgical, Inc., Sarasota, FL, USA). This ultrathin needle, specifically designed for delicate procedures involving the subretinal space, provides precise and controlled administration of medications. The cannula was loaded with a dose of 25–50 *μ*g tPA, sufficient to completely cover the area of the hemorrhage. The volume of the balanced salt solution was added at the surgeon's discretion. The primary purpose of the follow-up was to assess the patient's VA recovery at 1 and 3 months postoperative and to evaluate any complications associated related to the surgery. Patients who received intravitreal tPA injections and did not undergo surgery or had insufficient follow-up were excluded from the study. Notably, we used a new method of minimally invasive surgery, where we started pushing the syringe just as the cannula was about to touch the puncturing part of the retina. We also used multiple punctures for larger blood clots. In this case, when the cannula enters the retina, it immediately forms a barrier between the retinal neurosensory layer and the retinal pigment epithelium. This can prevent cannula damage to the retinal pigment epithelium or choroid layer during the procedure and even prevent the complication of choroidal rupture.

### 2.3. Statistical Analysis

Visual acuity was measured using the Snellen visual acuity (SVA) chart, and the SVA was analyzed using Wilcoxon-paired signed rank tests (SPSS v.22.0 software; IBM Corp., Armonk, NY, USA) preoperatively and at 1 and 3 months postoperatively. All SVA results were converted to logMAR for analysis. The VA test that involves counting fingers and hand motion yielded results of 1.85 and 2.3 logMAR, respectively. The significance level was set at 5%.

### 2.4. Ethical Considerations

This study was approved by the Ethics Committee of Wuhan University Aier Eye Hospital and conformed to the guiding principles of the Declaration of Helsinki [19]. Informed consent was obtained from all patients prior to undergoing PPV with subretinal tPA (PPV + tPA).

## 3. Results

### 3.1. Clinical Data of Patients with RAM and PCV

We reviewed the clinical data of 53 patients with SMH, 36 of whom met the criteria and had sufficient follow-up data. The specific admission test information is listed in Table 1.

### 3.2. Clinical Management of Patients with RAM and PCV

**Figure 1:**
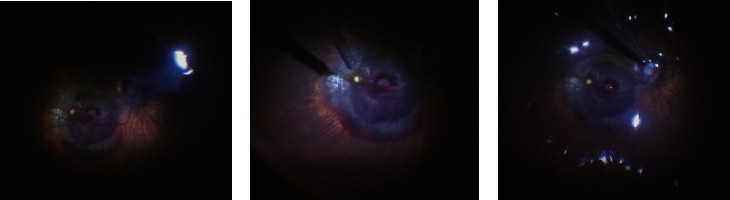
PPV + subretinal tPA injection + air tamponade surgery in patients with SMH.

The most common diagnosis was RAM (52.78%, 19/36) followed by PCV (47.22%, 17/36 patients). The median VA was 1.85 logMAR before surgery, 0.93 and 0.98 logMAR at 1 and 3 months after surgery, respectively. There was a significant difference in VA before and after surgery (*P* > 0.05), but no significant difference was shown in VA at 1 and 3 months after surgery (*P* > 0.05). All 36 patients underwent PPV + subretinal tPA injection + air tamponade, and OCT measurements and fundus photography were performed preoperatively and at 1 month postoperatively. The result shows a patient having SMH undergoing PPV + tPA surgery (as shown in Figure 1(a)). Red subretinal hemorrhage and retinal detachment are visible. The result shows a 38 G needle head injecting tPA diluent into the area below the retina (as shown in Figure 1(b)). The gas-liquid exchange happens in the vitreous cavity; the vitreous cavity is filled with filtered and sterilized air (as shown in Figure 1(c)).

### 3.3. PPV + tPA Had a Significant Positive Effect in Patients with SMH due to RAM and PCV

**Figure 2:**
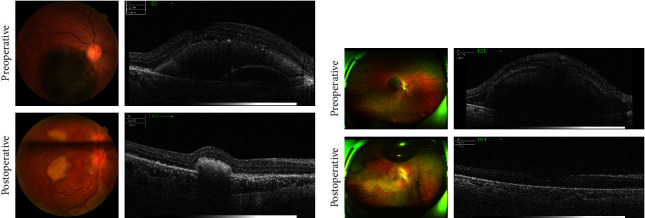
Comparison of fundus photographs and OCT in patients with SMH due to (a) PCV and (b) RAM before and after PPV + subretinal tPA injection + air tamponade surgery.

We performed fundus photography and OCT analysis in patients with PCV and RAM to compare the patient's status preoperatively and 1 month postoperatively. The preoperative fundus photograph of a patient with PCV showed a dark red blood clot under the macular retina with a bulge of the retina. One month after the operation, the upper vitreous cavity was partially filled with air, and the dark red blood clot under the macular retina dissolved, leaving only a yellowish white scar. Preoperative OCT results showed preoperative hemorrhage involving the macula, and there were hemorrhagic bulges under the neuroepithelium and under the pigment epithelium in the fovea. One month after the operation, OCT results showed that the red blood clot had migrated from the macular subretinal area leaving an old subretinal scar (as shown in Figure 2(a)). The preoperative fundus photograph of a patient with RAM showed extensive subretinal hemorrhage in the macular area, severe retinal bulge, and visible exudation around the blood clot. One month after surgery, there was subretinal hematocele dispersed in the macular area, but no retinal detachment was observed (as shown in Figure 2(b). Preoperative OCT results showed subretinal hemorrhage, which had accumulated into the foveomacula, and visible detachment of the macular retinal neurosensory layer. After surgery, the air injected into the vitreous cavity, for the purposes of adhesion formation between the retinal neurosensory layer and the retinal pigment epithelium, had completely absorbed, and the submacular hematocele in the macular retina had been dispersed (as shown in Figure 2(b)).

We followed up the 36 patients and recorded the BCVA and any complications at 1 and 3 months postoperatively. The follow-up information is listed in Table 2.

### 3.4. Postoperative Visual Acuity Was Improved

In conclusion, 32/36 patients (88.89%) experienced visual improvement postoperatively, with 15/36 patients (41.67%) gaining 3+ lines of Snellen acuity and 17/36 patients (47.22%) gaining 1-2 lines of Snellen acuity improvement. Four patients showed no visual improvement. There was improved VA in patients with PCV (94.12%) and RAM (84.21%). One month after the operation, only patient no. 18 was diagnosed with rhegmatogenous retinal detachment (RRD). Of the 36 patients followed up 3 months later, 4 had VH and 1 had symptoms of RRD. Four patients had no improvement in vision after 1 month. In Patient 3 with severe cataract (a severely opacified lens nucleus, an opacified cortical wheel in more than 2 quadrants, and >50% posterior capsular opacification), phacoemulsification was combined with PPV + tPA surgery. Twelve patients had worse VA at the postoperative 3 months follow-up than at postoperative 1 month, including six patients with PCV and six patients with RAM. Possible reasons for this decline could be surgical complications or the natural progression of their disease. All patients underwent PPV + subretinal tPA injection + air tamponade. One patient was additionally treated with phacoemulsification.

## 4. Discussion

SMH is a devastating complication of neovascular age-related macular degeneration [20] with multiple underlying disease processes that can lead to acute episodes and even dense submacular bleeding in the fovea resulting in severe vision loss if left untreated [8]. In recent years, the treatment of SMH has improved significantly owing to widespread use of anti-VEGF drugs such as ranibizumab and improved surgical methods [21]. Current treatment options can be divided into intravitreous injection alone and vitrectomy combined with intravitreous and/or subretinal injection. The least invasive of the treatment regimens involves the intravitreous injection of anti-VEGF agents, tPA, and/or expandable gas aimed at treating potential choroidal neovascularization and/or displacing submacular blood [22]. Furthermore, studies have shown that PPV + tPA and air tamponade result in moderate visual recovery in patients with SMH, PCV [23, 24], and RAM [25]. Therefore, none of the patients in this study received any relevant ocular treatment prior to surgery, nor did they receive intravitreal injections of anti-VEGF drugs during the subsequent observation period.

PPV + subretinal tPA injection + air tamponade was used in this study to evaluate SVA and SMH anatomical displacement and surgical complications [26, 27]. As an enzyme, tPA has the unique ability to convert plasminogen into plasmin, a proteolytic enzyme that breaks down blood clots by degrading fibrin, a protein integral to clot formation. By doing so, tPA essentially liquifies the blood clot, reducing its density and cohesiveness. In terms of surgical treatment details, we start to push the syringe just before the cannula touches the puncture part of the retina, so as to avoid additional damage to the retina by the tip of the cannula. Large clots require multiple punctures to reduce the risk of complications associated with clot removal. The formation of a vacuole via this injection method serves a critical function in the surgical process. The vacuole swiftly coats the surface of the blood clot, a feature that enhances the likelihood of surgical success and has prompted widespread adoption of this technique in our hospital following the subretinal tPA injection; an air tamponade is introduced into the vitreous cavity. The buoyant force of the air bubble helps to displace the liquified blood clot from the macula to a peripheral location, thus reducing the impact on central vision. Our results showed that PPV combined with subretinal tPA and air tamponade improved VA in patients with SMH, regardless of PCV or RAM. OCT results showed that in most patients after treatment, the hematoceles and exudates under the neuro- and pigmented epithelium of the macular retina had dispersed and had been resolved, no retinal detachment was seen, and the mild or severe retinal humps had been restored, which are all signs of visual improvement. In this study, patients with PCV and RAM had postoperative complications (VH and RRD), and 12 patients had worsening VA at 3 months after surgery compared with 1 month after surgery, which is consistent with other studies [7, 28], that is, VA loss in PCV and RAM patients may worsen with disease course. Meanwhile, there was no choroidal rupture as a postoperative complication, which may be a positive effect of our slightly innovative surgical approach.

All 36 patients were followed up for more than 3 months. Given that many of these patients might have continued to gradually lose their vision because of the natural course of the disease, BCVA at the 1 and 3 months follow-up was analyzed as the primary outcome only when evaluating the outcome of surgical interventions. We achieved modest vision recovery in 88.89% of the patients. The incidence of postoperative complications, including VH and RRD, was comparable or lower than those previously reported. We did not witness complications such as anterior chamber hemorrhage and cataract which have been observed in other studies [29, 30]. Studies have shown that patients who continue to receive anti-VEGF therapy after surgery have improved the prognosis, but it is not clear whether further vision recovery can be achieved after continuous treatment of dense subretinal macular hemorrhage [31]. This suggests that we should be cautious in continuing anti-VEGF therapy in patients with SMH after surgery. Meanwhile, the correlation between early treatment and improved visual outcomes remains to be determined.

Our study had several limitations. First, the number of patients was too small for the results to be considered statistically significant. Second, the follow-up of some patients with complications was not included in the analysis due to patient discontinuation, which affects the completeness and comprehensiveness of the data. Finally, the postoperative follow-up period may have been too short, and a longer follow-up period may be needed to evaluate the possible causes of late visual impairment.

## 5. Conclusion

In the short term, PPV with subretinal injection of tPA and air tamponade administered to patients with SMH, including patients with PCV and RAM, led to modest visual improvement in 88.89% patients, although long-term maintenance of VA may depend on the underlying pathological condition. Adverse events occurred in 11.11% patients in the cohort, raising concerns regarding the importance of careful patient selection. Thus, when obtaining informed consent, the potential benefits and potential risks of the procedure must be carefully discussed with the patient. Patient selection could consider factors such as age, overall health, underlying conditions, and potential for the procedure to improve the specific eye condition.

## Figures and Tables

**Table 1:** Patient clinical data.

Patient	Age (year)	Sex	Duration (day)	Lens state	Preop VH	Preop BCVA (logMAR)	Diagnosis
1	70	M	30	C2N1P1	+	1.85	RAM
2	65	F	1	C1N1P1	−	1.85	RAM
3	78	F	4	C3N3P3	−	1.85	RAM
4	60	M	20	C1N1P0	+	2.3	PCV
5	79	F	14	IOL	+	1.85	RAM
6	46	M	30	C0N1P0	−	2.3	PCV
7	61	M	10	C1N1P1	−	0.8	PCV
8	64	F	14	C1N1P1	−	1.85	RAM
9	64	M	15	C1N1P0	+	2.3	PCV
10	76	M	15	IOL	+	1.6	RAM
11	52	M	15	C1N1P0	−	0.7	PCV
12	67	F	7	C2N1P1	+	1.85	RAM
13	73	F	31	C1N1P0	+	1.85	PCV
14	55	M	20	C2N1P1	+	2.3	PCV
15	75	F	33	IOL	+	1.2	RAM
16	62	M	3	C1N1P1	+	1.85	RAM
17	56	F	20	C1N1P0	−	1.0	PCV
18	58	F	97	C1N1P1	+	2.3	PCV
19	62	F	99	C1N1P1	+	2.3	PCV
20	47	M	35	C1N1P1	+	2.3	RAM
21	68	F	91	C3N1P1	+	2.3	PCV
22	61	F	20	C2N1P1	+	1.85	PCV
23	60	F	4	IOL	−	1.2	PCV
24	65	F	127	C2N2P1	−	1.85	RAM
25	63	F	1	C1N1P1	−	0.8	RAM
26	75	F	66	C2N2P1	+	1.85	RAM
27	60	F	56	C1N1P1	+	1.85	PCV
28	75	M	28	C2N1P1	+	1.85	PCV
29	60	M	44	C1N1P1	−	1.85	RAM
30	57	M	67	C2N1P1	−	1.2	RAM
31	61	F	90	C1N1P0	+	2.3	PCV
32	75	M	55	C3N3P3	+	1.0	RAM
33	68	F	28	C2N1P1	+	2.3	PCV
34	73	F	56	C3N1P1	−	1.2	RAM
35	53	F	68	C3N1P1	+	1.85	RAM
36	60	M	65	C1N1P1	+	1.85	RAM

F: female; M: male; duration: interval from the initial symptom or decreased vision to the time of the surgery; VH: vitreous hemorrhage; BCVA: best-corrected visual acuity; preop: preoperative; SMH: submacular hemorrhage; PCV: polypoidal choroidal vasculopathy; RAM: retinal arterial macroaneurysm; IOL: intraocular lens; N: the degree of nuclear opacity of the lens. The criteria are as follows: 0: transparency; 1: early turbidity; 2: medium turbidity; 3: severe turbidity. C: opacity of the lens cortex. The criteria are as follows: 0: transparency; 1: a small amount of spotty turbidity; 2: the punctate turbidity is enlarged, and a small amount of punctate turbidity appears in the pupil area; 3: wheel-like turbidity, more than 2 quadrants. P: degree of subcapsular opacification of posterior lens. The criteria are as follows: 0: transparency; 1: about 3% turbidity; 2: about 30% turbidity; 3: about 50% turbidity.

**Table 2:** Patients' follow-up data.

Patient	Surgical form	BCVA at postop 1 month	BCVA at postop 3 months	Postop 1 month	Postop 3 months
				Complications	Complications
1	PPV + tPA	0.5	0.5	—	—
2	PPV + tPA	0.5	0.5	—	—
3	Phaco + PPV + tPA	1.85	1.85	—	—
4	PPV + tPA	0.4	0.5	—	—
5	PPV + tPA	0.7	0.9	—	—
6	PPV + tPA	0.6	0.6	—	—
7	PPV + tPA	0.4	0.6	—	—
8	PPV + tPA	0.6	0.6	—	—
9	PPV + tPA	0.8	0.8	—	—
10	PPV + tPA	0.8	0.9	—	—
11	PPV + tPA	0.5	0.5	—	—
12	PPV + tPA	0.5	0.5	—	—
13	PPV + tPA	1	1.2	—	—
14	PPV + tPA	0.6	0.6	—	—
15	PPV + tPA	0.4	0.4	—	—
16	PPV + tPA	0.4	0.6	—	VH
17	PPV + tPA	0.3	0.4	—	—
18	PPV + tPA	2.3	2.3	RRD	RRD
19	PPV + tPA	1.85	1.85	—	—
20	PPV + tPA	1.85	1.85	—	VH
21	PPV + tPA	1.85	1.85	—	VH
22	PPV + tPA	0.8	1.0	—	—
23	PPV + tPA	0.9	0.9	—	—
24	PPV + tPA	1.85	1.85	—	—
25	PPV + tPA	0.3	0.4	—	—
26	PPV + tPA	1.85	1.85	—	—
27	PPV + tPA	0.6	0.8	—	—
28	PPV + tPA	0.5	0.5	—	—
29	PPV + tPA	0.6	0.6	—	—
30	PPV + tPA	0.3	0.4	—	—
31	PPV + tPA	1.85	1.85	—	VH
32	PPV + tPA	0.4	0.4	—	—
33	PPV + tPA	1.85	1.85	—	RRD
34	PPV + tPA	0.5	0.5	—	—
35	PPV + tPA	0.7	0.9	—	—
36	PPV + tPA	1	1.2	—	—

VH: vitreous hemorrhage; BCVA: best-corrected visual acuity; postop: postoperative; Phaco: phacoemulsification; tPA: tissue plasminogen activator; PPV: pars plana vitrectomy; RRD: rhegmatogenous retinal detachment.

## Data Availability

The data used to support the findings of this study are available from the corresponding author upon reasonable request.
